# Transient Pseudohypoaldosteronism Secondary to Group B Streptococcus Pyelonephritis

**DOI:** 10.7759/cureus.15071

**Published:** 2021-05-17

**Authors:** Atsuo Morisaki, Yuki Naruse, Yui Shibata, Masato Mori, Ryugo Hiramoto

**Affiliations:** 1 Pediatrics, Children Center, Matsudo City General Hospital, Matsudo, JPN

**Keywords:** group b streptococcus, pseudohypoaldosteronism, hyperkalemia, urinary tract malformations, urinary tract infections

## Abstract

Secondary pseudohypoaldosteronism is a condition characterized by aldosterone resistance in renal tubules. It is highly associated with urinary tract infection and urinary tract malformations. Only a few cases of pseudohypoaldosteronism secondary to group B *Streptococcus *pyelonephritis have been reported to date. A four-month-old boy developed poor sucking and weight loss, and his laboratory test results revealed hyponatremia, hyperkalemia, renal dysfunction, high anion gap metabolic acidosis, pyuria, and hydronephrosis. Laboratory tests including urinalysis confirmed the diagnosis of pseudohypoaldosteronism secondary to group B *Streptococcus*. He was treated with intravenous normal saline and antimicrobial therapy. Electrolyte disorders were addressed and he was discharged on the 10th day of hospitalization without any sequelae. Voiding cystourethrography performed after discharge showed bilateral grade 5 vesicoureteral reflux and intrarenal reflux in the right kidney. Transient pseudohypoaldosteronism is an important consideration in the differential diagnosis in infants with hyponatremia and hyperkalemia. A thorough evaluation for urinary tract malformations should be performed, including early abdominal ultrasonography and systemic management.

## Introduction

Type 1 pseudohypoaldosteronism is a condition characterized by hyponatremia, hyperkalemia, and metabolic acidosis. It is associated with aldosterone resistance in renal tubules. The syndrome is subdivided into a genetic form and secondary form. Secondary pseudohypoaldosteronism is known to be associated with urinary tract malformations and urinary tract infections [1,2]. *Escherichia coli* is the most common pathogen causing urinary tract infections in patients with secondary pseudohypoaldosteronism [2]. Only a few cases of pseudohypoaldosteronism secondary to group B *Streptococcus* (GBS) infection have been reported to date. We report a case of a four-month-old boy who developed poor sucking and weight loss and was diagnosed with GBS pyelonephritis and a urinary tract malformation.

## Case presentation

A previously healthy four-month-old male infant was referred and subsequently admitted to our hospital with poor sucking that started two days prior to presentation. He was born healthy at 40 weeks and 0 days of gestation by normal vaginal delivery. There was no history of any maternal infection. His birth weight was 3454 g, which was appropriate for his gestational age. He had been exclusively breastfed and was well with a weight of 6300 g at his four-month supervision one week prior to admission. On admission, he was 63.6 cm (-0.26 SD) in length and 5670 g in weight, which was 10% less than his weight from the previous examination. His vital signs were stable except for mild hypotension (blood pressure, 69/48 mmHg; heart rate, 122 bpm; respiratory rate, 34/min; body temperature. 36.7℃; and SpO_2_ at room air, 100%). On physical examination, facial pallor, sunken eyes and fontanel, dry mucous membranes, mild delay in skin elasticity, delayed capillary refill (2 s), reticular cyanosis, and coldness of extremities were noted. Cardiac and respiratory examination findings were unremarkable. There were no external genital malformations, and external genital or general skin pigmentation.

Blood test results (Table 1) showed hyponatremia (serum Na+, 114 mEq/L), hyperkalemia (serum K+, 9.3 mEq/L), renal dysfunction, and high anion gap metabolic acidosis. There was no hypoglycemia. Inflammatory markers were not elevated. Urinalysis revealed pyuria, hematuria, and proteinuria. Chest and abdominal x-rays revealed no abnormalities. Ultrasonography showed no abnormalities in the head or heart but revealed left hydronephrosis (grade unknown). Electrocardiography showed peaked T waves but no QT prolongation (QTc, 437 ms).

**Table 1:** Blood Test Results WBC: white blood cells; N: neutrophil; L: lymphocyte; RBC: red blood cells; Hb: hemoglobin; Hct: hematocrit; Plt: platelets; BE: base excess; Lac: lactate; Glu: glucose; AST: aspartate transaminase; ALT: alanine aminotransferase; LDH: lactate dehydrogenase; BUN: blood urea nitrogen; Cre: creatinine; CRP: C-reactive protein; ACTH: adrenocorticotropin; PRA: plasma renin activity; PAC: plasma aldosterone concentration

Test	Value		Normal Range	Test	Value		Normal Range
Complete Blood Count				Biochemistry			
WBC	20,000	/μL	(4600-18,900)	AST	88	U/L	(22-66)
N	41	%	(20-50)	ALT	79	U/L	(13-56)
L	52	%	(44-74)	LDH	249	U/L	(205-418)
RBC	507	10^4^/μL	(340-500)	BUN	66.4	mg/dL	(2.2-14.1)
Hb	12.9	g/dL	(9.5-13.7)	Cre	0.96	mg/dL	(0.12-0.27)
Hct	38.8	%	(28.5-41.1)	Na	114	mEq/L	(135-143)
Plt	77.3	10^3^/μL	(25.0-82.0)	Cl	88	mEq/L	(101-111)
				K	9.3	mEq/L	(4.1-5.6)
Blood Gas (Vein)				CRP	0.69	mg/dL	(<0.30)
pH	7.178		(7.36-7.44)				
pCO_2_	25.6	mmHg	(36-44)	Endocrine Examination			
HCO_3_^-^	9.3	mmol/L	(22-26)	ACTH	6.6	pg/mL	(11.6-35.0)
BE	-17.4	mmol/L	(-2〜+3)	Cortisol	47.3	μg/dL	(3.0-23.0)
Lac	4.6	mmol/L	(<2.0)	PRA	>20	ng/mL/h	(0.3-2.9)
Glu	160	mg/dL	(50-109)	PAC	42,700	pg/mL	(35.7-240)

Aldosterone deficiency was suspected owing to the presence of hyponatremia, hyperkalemia, metabolic acidosis, and dehydration. His plasma aldosterone concentration was elevated, which ruled out aldosterone deficiency. Adrenal insufficiency was also considered in the differential diagnosis but was less likely because of the absence of hypoglycemia. The patient was diagnosed with secondary pseudohypoaldosteronism owing to his high plasma renin activity and high serum aldosterone levels in his admission samples. Adrenocorticotropin (ACTH) assay was within normal ranges and serum cortisol assay was elevated, which excluded adrenal insufficiency. Cortisol replacement was not performed. Electrolyte abnormalities were corrected with rapid infusion of normal saline, glucose-insulin therapy, intravenous calcium gluconate hydrate, sodium bicarbonate, and oral cation exchange resin. Electrolyte abnormalities improved within 4 h of admission.

The patient did not have any episodes of fever; however, he was treated with cefotaxime 200 mg/kg/day and ampicillin (ABPC) 200 mg/kg/day because of leukocytosis and pyuria. Although abdominal ultrasonography on admission showed left hydronephrosis, no hydronephrosis was found in the repeat examination on the second day of admission. On the fifth day of hospitalization, GBS was detected in the urine culture at a concentration of 10^8^/mL, and the antimicrobial agent was changed to ABPC monotherapy. Blood culture was sterile. He was discharged on the 10th day of hospitalization.

**Figure 1:**
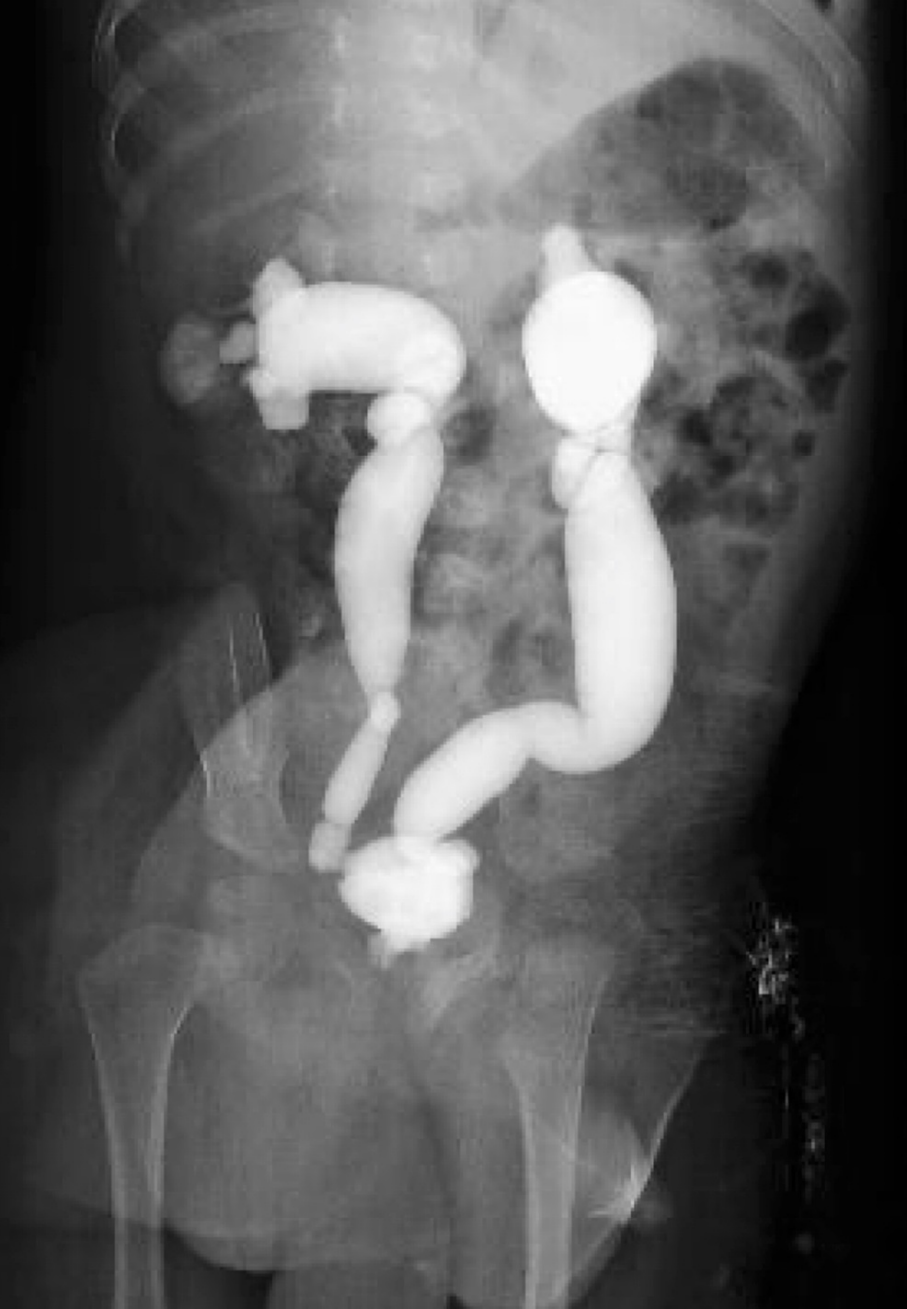
Voiding Cystourethrogram Voiding cystourethrogram revealed bilateral grade 5 vesicoureteral reflux and intrarenal reflux in the right kidney

After discharge from the hospital, voiding cystourethrogram (Figure 1) was performed, which revealed bilateral grade 5 vesicoureteral reflux and intrarenal reflux in the right kidney. On the 28th day, plasma renin activity and serum aldosterone levels improved to 4.0 ng/mL/h and 679 pg/mL, respectively. Two months after discharge from the hospital, ureteroneocystostomy was performed. The patient progressed without subsequent recurrence of urinary tract infection. At the age of two years and five months, he was 92.0 cm (+0.96 SD) in height and 12.7 kg (+0.94 SD) in weight, with good growth and normal neurodevelopment.

## Discussion

Since Rodríguez-Soriano et al. reported the first pediatric case of secondary pseudohypoaldosteronism in 1983 [3], more than 100 cases have been reported. Secondary pseudohypoaldosteronism mainly occurs in infants younger than three months and is highly associated with male gender, urinary tract infection, and urinary tract malformations [1]. In Delforge’s review of secondary pseudohypoaldosteronism caused by urinary tract malformations [4], the condition was more frequently associated with male gender (81.9%) and urinary tract infection (90.5%). Among urinary tract malformations, vesicoureteral reflux (47.4%) was more common.

GBS is an unusual urinary tract pathogen and is highly associated with urinary tract malformation in children [5,6]. Only a few cases of GBS-related pseudohypoaldosteronism have been reported to date [7-9]. GBS urinary tract infection in an infant may result from hematogenous seeding of bacteria [10]; however, the blood culture was sterile in our case. Therefore, our patient was diagnosed with an ascending urinary tract infection. According to the age of onset, GBS infections are classified as early-onset, late-onset, or late-late-onset disease (LLOD). Our patient’s infection was classified as LLOD because the onset started after the age of 89 days. LLOD accounts for 10% of all infantile GBS infections and typically occurs in infants who were delivered at less than 35 weeks of gestation [11]; however, our patient was delivered vaginally at full term and had no history of maternal infection.

Secondary pseudohypoaldosteronism mainly causes nonspecific symptoms, such as poor feeding, poor weight gain, vomiting, failure to thrive, and dehydration [1]. Some cases have reported convulsive seizures with sequelae, fatal arrhythmias, and cardiac arrest [12-14]. The symptoms resolve with intravenous fluids, electrolyte correction, and antibiotic therapy, and the prognosis is good. Therefore, early diagnosis is required to prevent serious complications and sequelae.

Congenital adrenocortical hyperplasia and congenital adrenal hypoplasia are considered in the differential diagnoses of salt-losing nephropathy accompanied by hyperkalemia [15]. Our patient had no external genital malformations, external genital or general skin pigmentation, or hypoglycemia; therefore, these conditions were unlikely. The patient had pyuria on urinalysis and hydronephrosis on ultrasonography and was treated according to the first diagnosis of secondary pseudohypoaldosteronism. After electrolyte correction on admission, the electrolytes remained stable. The patient was diagnosed with secondary pseudohypoaldosteronism based on the results of plasma renin, serum aldosterone, ACTH, and serum cortisol levels obtained at the time of admission. ACTH levels were slightly low but might have been caused by the time taken from sample collection to plasma separation. Adrenal insufficiency-related syndrome could have been included in the differential diagnosis based on the initial general condition of the patient. A therapeutic strategy of concomitant glucocorticoid supplementation could have been considered since cases of initial treatment with glucocorticoids have been reported [1,13].

The pathophysiology of secondary pseudohypoaldosteronism remains unclear. Approximately 80% of patients with transient pseudohypoaldosteronism are less than seven months old [16]; therefore, it is speculated that tubular immaturity may contribute to the pathogenesis of the disease because this would require high aldosterone levels to maintain electrolyte balance [4]. Infection and urinary tract obstruction are considered to cause increased aldosterone resistance in renal tubules, both directly through the impairment of cellular response mechanisms and indirectly through changes in renal hormone levels [17]. The onset of secondary pseudohypoaldosteronism occurred at four months of age in our case, suggesting that this condition should be considered in the differential diagnosis of hyponatremia and hyperkalemia in early infancy.

## Conclusions

A four-month-old boy who developed poor sucking and weight loss was diagnosed with pseudohypoaldosteronism secondary to GBS pyelonephritis. Pseudohypoaldosteronism is important in the differential diagnosis in early infants with hyponatremia and hyperkalemia. A thorough evaluation for urinary tract malformations should be performed, including abdominal ultrasonography at an early stage, alongside systemic management.
